# (2*Z*)-4-[(2-Hy­droxy­phen­yl)carbamo­yl]prop-2-enoic acid

**DOI:** 10.1107/S1600536810045496

**Published:** 2010-11-13

**Authors:** Farooq Ali Shah, Saqib Ali, M. Nawaz Tahir, Sajjad Ahmed

**Affiliations:** a Department of Chemistry, Quaid-e-Azam University, Islamabad, Pakistan; b Department of Physics, University of Sargodha, Sargodha, Pakistan

## Abstract

In the title compound, C_10_H_9_NO_4_, the 2-hy­droxy­anilinic and the 4-oxobut-2-enoic acid groups are almost planar, with r.m.s. deviations of 0.0086 and 0.0262 Å, respectively. The dihedral angle between the two groups is 6.65 (1)°. Intra­molecular N—H⋯O, C—H⋯O and O—H⋯O hydrogen bonds form *S*(5), *S*(6) and *S*(7) ring motifs. In the crystal, the mol­ecules are dimerized due to C—H⋯O and O—H⋯O inter­molecular hydrogen bonds, with *R*_2_^2^(8) ring motifs. The dimers are inter­linked into polymeric chains along [010] with *R*_4_^3^(13) ring motifs by C—H⋯O, N—H⋯O and O—H⋯O hydrogen bonds.

## Related literature

For background and a related structure, see: Shah *et al.* (2008 ▶). For graph-set notation, see: Bernstein *et al.* (1995 ▶).
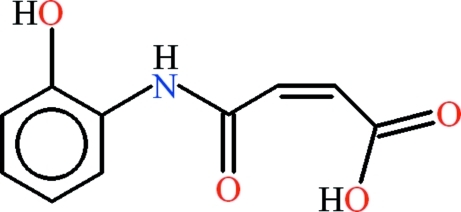


## Experimental

### 

#### Crystal data

C_10_H_9_NO_4_
*M* *_r_* = 207.18
Orthorhombic, 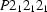

*a* = 6.7873 (3) Å
*b* = 10.6855 (4) Å
*c* = 12.8442 (4) Å
*V* = 931.54 (6) Å^3^
*Z* = 4
Mo *K*α radiation
μ = 0.12 mm^−1^
*T* = 296 K
0.32 × 0.25 × 0.24 mm

#### Data collection

Bruker Kappa APEXII CCD diffractometer
Absorption correction: multi-scan (*SADABS*; Bruker, 2005 ▶) *T*_min_ = 0.968, *T*_max_ = 0.978
4118 measured reflections
997 independent reflections
944 reflections with *I* > 2σ(*I*)
*R*_int_ = 0.015

#### Refinement

*R*[*F* ^2^ > 2σ(*F* ^2^)] = 0.030
*wR*(*F* ^2^) = 0.082
*S* = 1.09
997 reflections
138 parameters
H-atom parameters constrained
Δρ_max_ = 0.17 e Å^−3^
Δρ_min_ = −0.18 e Å^−3^

### 

Data collection: *APEX2* (Bruker, 2009 ▶); cell refinement: *SAINT* (Bruker, 2009 ▶); data reduction: *SAINT*; program(s) used to solve structure: *SHELXS97* (Sheldrick, 2008 ▶); program(s) used to refine structure: *SHELXL97* (Sheldrick, 2008 ▶); molecular graphics: *ORTEP-3 for Windows* (Farrugia, 1997 ▶) and *PLATON* (Spek, 2009 ▶); software used to prepare material for publication: *WinGX* (Farrugia, 1999 ▶) and *PLATON*.

## Supplementary Material

Crystal structure: contains datablocks global, I. DOI: 10.1107/S1600536810045496/bq2250sup1.cif

Structure factors: contains datablocks I. DOI: 10.1107/S1600536810045496/bq2250Isup2.hkl

Additional supplementary materials:  crystallographic information; 3D view; checkCIF report

## Figures and Tables

**Table 1:** Hydrogen-bond geometry (Å, °)

*D*—H⋯*A*	*D*—H	H⋯*A*	*D*⋯*A*	*D*—H⋯*A*
N1—H1⋯O1	0.86	2.20	2.6111 (19)	109
O1—H1*A*⋯O4^i^	0.82	1.94	2.7282 (18)	162
O3—H3*A*⋯O2	0.82	1.67	2.4881 (18)	175
C3—H3⋯O3^i^	0.93	2.47	3.374 (2)	164
C6—H6⋯O2	0.93	2.26	2.850 (2)	121
C8—H8⋯O4^ii^	0.93	2.57	3.404 (2)	150

Symmetry codes: (i) 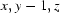; (ii) 
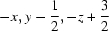
.

**Fig. 1.**
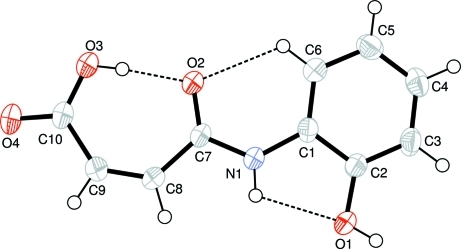
View of the title compound with the atom numbering scheme. The thermal displacements are drawn at the 50% probability level. The dotted line indicate the intramolecular H-bond.

**Fig. 2.**
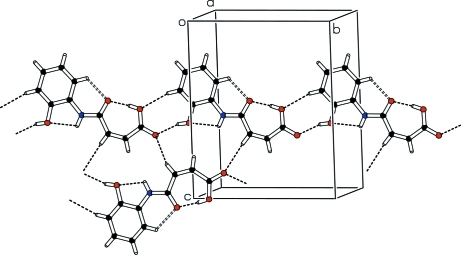
The partial packing (PLATON; Spek, 2009) which shows that molecules form one-dimensional polymeric network due to strong intermolecular H-bondings.

## supplementary crystallographic information

### Comment

We reported the crystal structure of (II) *i.e.*, 4-[(2-Fluorophenyl)amino]-4-oxobutanoic acid (Shah *et al.*, 2008), previously. The title compound (I, Fig. 1), has been prepared in continuation to synthesize substituted oxobutanoic acids.

In (I), the 2-hydroxyanilinic group (C1—C6/O1/N1) and the other part *i.e.*, 4-oxobut-2-enoic acid (C7—C10/O2/O3/O4) are planar with r.m.s. deviation of 0.0086 Å and 0.0262 Å, respectively. The dihedral angle between two groups is 6.65 (1)°. The intramolecular H-bondings of N—H···O, C—H···O and O—H···O types (Table 1, Fig. 1) complete S(5), S(6) and S(7) ring motifs (Bernstein *et al.*, 1995). Generally, the carboxylic acids are dimerized through O—H···O bonds with *R*_2_^2^(8) ring motifs but the molecules of (I) are dimerized in a different way. The molecules of (I) are dimerized due to C—H···O and O—H···O intermolecular H-bondings with *R*_2_^2^(8) ring motifs (Table 1, Fig. 2). In these dimers the donor and accepter groups are from separate molecules. This change has been occurred due to the attachment of hydroxy group at the position-2 of benzene ring and the presence of carbonyl group. The dimers are interlinked in the form of polymeric chains extending along the crystallographic b-axis *i.e.*, along [010] with *R*_4_^3^(13) ring motifs due to C—H···O, N—H···O and O—H···O tyeps of H-bondings (Table 1, Fig. 2).

### Experimental

2-Hydroxyaniline (10.9 g, 0.1 mol) was dissolved in 30 ml of glacial acetic acid. A solution of Maleic anhydride (9.8 g, 0.1 mol) in 50 ml glacial acetic acid was added and the mixture was stirred overnight. The precipitate which appeared were filtered, washed with distilled water and dried at 313–315 K. The acid was recrystallized from acetone to get yellow prisms of title compound. (Yield: 85%, m.p. 415 K)

### Refinement

The H-atoms were positioned geometrically with (O—H = 0.82, N—H = 0.86, C–H = 0.93 Å) and were included in the refinement in the riding model approximation, with *U*_iso_(H) = *xU*_eq_(C, N, O), where *x* = 1.2 for all H-atoms.

### Crystal data

**Table:**

C_10_H_9_NO_4_	*F*(000) = 432
*M**_r_* = 207.18	*D*_x_ = 1.477 Mg m^−^^3^
Orthorhombic, *P*2_1_2_1_2_1_	Mo *K*α radiation, λ = 0.71073 Å
Hall symbol: P 2ac 2ab	Cell parameters from 944 reflections
*a* = 6.7873 (3) Å	θ = 3.2–25.2°
*b* = 10.6855 (4) Å	µ = 0.12 mm^−^^1^
*c* = 12.8442 (4) Å	*T* = 296 K
*V* = 931.54 (6) Å^3^	Prism, yellow
*Z* = 4	0.32 × 0.25 × 0.24 mm

### Data collection

**Table:**

Bruker Kappa APEXII CCD diffractometer	997 independent reflections
Radiation source: fine-focus sealed tube	944 reflections with *I* > 2σ(*I*)
graphite	*R*_int_ = 0.015
Detector resolution: 8.2 pixels mm^-1^	θ_max_ = 25.2°, θ_min_ = 3.2°
ω scans	*h* = −7→8
Absorption correction: multi-scan (*SADABS*; Bruker, 2005)	*k* = −12→12
*T*_min_ = 0.968, *T*_max_ = 0.978	*l* = −15→14
4118 measured reflections	

### Refinement

**Table:**

Refinement on *F*^2^	Primary atom site location: structure-invariant direct methods
Least-squares matrix: full	Secondary atom site location: difference Fourier map
*R*[*F*^2^ > 2σ(*F*^2^)] = 0.030	Hydrogen site location: inferred from neighbouring sites
*wR*(*F*^2^) = 0.082	H-atom parameters constrained
*S* = 1.09	*w* = 1/[σ^2^(*F*_o_^2^) + (0.050*P*)^2^ + 0.1393*P*] where *P* = (*F*_o_^2^ + 2*F*_c_^2^)/3
997 reflections	(Δ/σ)_max_ < 0.001
138 parameters	Δρ_max_ = 0.17 e Å^−^^3^
0 restraints	Δρ_min_ = −0.18 e Å^−^^3^

### Special details

**Table:**

Geometry. Bond distances, angles *etc*. have been calculated using the rounded fractional coordinates. All su's are estimated from the variances of the (full) variance-covariance matrix. The cell e.s.d.'s are taken into account in the estimation of distances, angles and torsion angles
Refinement. Refinement of *F*^2^ against ALL reflections. The weighted *R*-factor *wR* and goodness of fit *S* are based on *F*^2^, conventional *R*-factors *R* are based on *F*, with *F* set to zero for negative *F*^2^. The threshold expression of *F*^2^ > σ(*F*^2^) is used only for calculating *R*-factors(gt) *etc*. and is not relevant to the choice of reflections for refinement. *R*-factors based on *F*^2^ are statistically about twice as large as those based on *F*, and *R*- factors based on ALL data will be even larger.

### Fractional atomic coordinates and isotropic or equivalent isotropic displacement parameters (Å^2^)

**Table:**

	*x*	*y*	*z*	*U*_iso_*/*U*_eq_	
O1	0.0644 (3)	−0.02680 (12)	0.57792 (9)	0.0460 (5)	
O2	0.0945 (3)	0.39908 (12)	0.45571 (10)	0.0498 (5)	
O3	0.0839 (4)	0.62508 (12)	0.50201 (11)	0.0565 (6)	
O4	0.0659 (3)	0.73178 (12)	0.64703 (12)	0.0519 (5)	
N1	0.0940 (3)	0.21180 (13)	0.53692 (11)	0.0339 (4)	
C1	0.0933 (3)	0.12928 (16)	0.45057 (14)	0.0311 (5)	
C2	0.0788 (3)	0.00188 (16)	0.47494 (13)	0.0333 (5)	
C3	0.0808 (3)	−0.08600 (17)	0.39600 (15)	0.0397 (6)	
C4	0.0939 (4)	−0.04766 (19)	0.29330 (16)	0.0416 (6)	
C5	0.1054 (3)	0.07754 (19)	0.26907 (14)	0.0403 (6)	
C6	0.1071 (3)	0.16658 (18)	0.34720 (14)	0.0358 (5)	
C7	0.0946 (3)	0.33779 (16)	0.53732 (14)	0.0312 (5)	
C8	0.0967 (3)	0.39436 (16)	0.64192 (14)	0.0341 (5)	
C9	0.0918 (3)	0.51549 (17)	0.66740 (14)	0.0366 (5)	
C10	0.0796 (3)	0.63080 (16)	0.60326 (15)	0.0384 (6)	
H1	0.09395	0.17669	0.59717	0.0407*	
H1A	0.06683	−0.10302	0.58522	0.0552*	
H3	0.07345	−0.17079	0.41187	0.0476*	
H3A	0.09210	0.55176	0.48367	0.0677*	
H4	0.09499	−0.10695	0.24030	0.0499*	
H5	0.11194	0.10243	0.19978	0.0483*	
H6	0.11753	0.25104	0.33063	0.0430*	
H8	0.10215	0.33850	0.69736	0.0409*	
H9	0.09700	0.53039	0.73868	0.0439*	

### Atomic displacement parameters (Å^2^)

**Table:**

	*U*^11^	*U*^22^	*U*^33^	*U*^12^	*U*^13^	*U*^23^
O1	0.0805 (11)	0.0226 (6)	0.0350 (7)	0.0016 (8)	−0.0057 (7)	0.0025 (5)
O2	0.0934 (12)	0.0237 (6)	0.0322 (7)	0.0007 (8)	−0.0019 (10)	0.0017 (5)
O3	0.1077 (14)	0.0221 (7)	0.0396 (8)	0.0033 (10)	−0.0012 (10)	0.0015 (5)
O4	0.0801 (11)	0.0239 (6)	0.0518 (9)	−0.0005 (8)	−0.0008 (9)	−0.0067 (6)
N1	0.0494 (9)	0.0225 (7)	0.0299 (7)	0.0008 (8)	−0.0004 (9)	0.0006 (5)
C1	0.0335 (9)	0.0249 (9)	0.0350 (9)	0.0011 (8)	−0.0023 (10)	−0.0039 (7)
C2	0.0402 (9)	0.0262 (9)	0.0335 (9)	0.0014 (9)	−0.0038 (9)	0.0005 (7)
C3	0.0480 (11)	0.0256 (9)	0.0455 (11)	−0.0017 (10)	−0.0046 (11)	−0.0065 (8)
C4	0.0467 (12)	0.0376 (10)	0.0404 (10)	−0.0027 (10)	−0.0003 (10)	−0.0120 (8)
C5	0.0449 (12)	0.0440 (11)	0.0319 (9)	−0.0015 (11)	−0.0008 (10)	−0.0026 (8)
C6	0.0426 (10)	0.0302 (9)	0.0347 (9)	0.0011 (9)	0.0009 (10)	0.0018 (8)
C7	0.0374 (9)	0.0213 (8)	0.0348 (9)	0.0002 (8)	−0.0006 (10)	0.0014 (7)
C8	0.0446 (11)	0.0257 (8)	0.0319 (9)	−0.0006 (10)	0.0016 (10)	0.0040 (7)
C9	0.0466 (10)	0.0314 (9)	0.0318 (9)	−0.0016 (10)	0.0028 (10)	−0.0031 (7)
C10	0.0481 (12)	0.0245 (9)	0.0427 (10)	−0.0014 (10)	−0.0009 (11)	−0.0025 (8)

### Geometric parameters (Å, °)

**Table:**

O1—C2	1.361 (2)	C3—C4	1.384 (3)
O2—C7	1.236 (2)	C4—C5	1.376 (3)
O3—C10	1.302 (2)	C5—C6	1.383 (3)
O4—C10	1.220 (2)	C7—C8	1.473 (3)
O1—H1A	0.8200	C8—C9	1.336 (2)
O3—H3A	0.8200	C9—C10	1.485 (3)
N1—C1	1.417 (2)	C3—H3	0.9300
N1—C7	1.346 (2)	C4—H4	0.9300
N1—H1	0.8600	C5—H5	0.9300
C1—C2	1.400 (2)	C6—H6	0.9300
C1—C6	1.389 (3)	C8—H8	0.9300
C2—C3	1.382 (3)	C9—H9	0.9300
			
O1···O4^i^	2.7282 (18)	C7···C1^viii^	3.424 (3)
O1···N1	2.6111 (19)	C8···C6^ix^	3.528 (3)
O1···C5^ii^	3.368 (3)	C8···C1^ix^	3.583 (3)
O2···C10	3.120 (2)	C8···C6^viii^	3.389 (3)
O2···C6	2.850 (2)	C8···C5^viii^	3.538 (3)
O2···O3	2.4881 (18)	C8···O4^vi^	3.404 (2)
O3···O2	2.4881 (18)	C9···C5^viii^	3.543 (3)
O3···C3^iii^	3.374 (2)	C9···C4^viii^	3.434 (3)
O3···C7	3.104 (2)	C9···C4^ix^	3.462 (3)
O4···C8^iv^	3.404 (2)	C9···C3^ix^	3.500 (3)
O4···C5^v^	3.404 (3)	C10···C3^viii^	3.419 (3)
O4···O1^iii^	2.7282 (18)	C10···O2	3.120 (2)
O1···H1	2.2000	C10···C3^ix^	3.435 (3)
O1···H5^ii^	2.8200	C4···H6^xii^	3.0400
O1···H9^vi^	2.6700	C6···H4^vii^	3.0000
O2···H6	2.2600	C7···H3A	2.3900
O2···H4^vii^	2.8300	C7···H6	2.8200
O2···H3A	1.6700	C8···H3A	2.6400
O3···H3^iii^	2.4700	C10···H1A^iii^	2.8600
O4···H5^v^	2.8900	H1···O1	2.2000
O4···H1A^iii^	1.9400	H1···H8	2.1600
O4···H8^iv^	2.5700	H1A···O4^i^	1.9400
N1···O1	2.6111 (19)	H1A···C10^i^	2.8600
C1···C7^viii^	3.407 (3)	H1A···H3	2.3400
C1···C7^ix^	3.424 (3)	H3···O3^i^	2.4700
C1···C8^viii^	3.583 (3)	H3···H1A	2.3400
C3···O3^i^	3.374 (2)	H3A···O2	1.6700
C3···C9^viii^	3.500 (3)	H3A···C7	2.3900
C3···C10^viii^	3.435 (3)	H3A···C8	2.6400
C3···C10^ix^	3.419 (3)	H4···O2^xii^	2.8300
C4···C9^viii^	3.462 (3)	H4···C6^xii^	3.0000
C4···C9^ix^	3.434 (3)	H4···H6^xii^	2.2800
C5···C8^ix^	3.538 (3)	H5···O1^x^	2.8200
C5···O1^x^	3.368 (3)	H5···O4^xi^	2.8900
C5···O4^xi^	3.404 (3)	H6···O2	2.2600
C5···C9^ix^	3.543 (3)	H6···C7	2.8200
C6···C8^ix^	3.389 (3)	H6···C4^vii^	3.0400
C6···O2	2.850 (2)	H6···H4^vii^	2.2800
C6···C8^viii^	3.528 (3)	H8···H1	2.1600
C7···C1^ix^	3.407 (3)	H8···O4^vi^	2.5700
C7···O3	3.104 (2)	H9···O1^iv^	2.6700
			
C2—O1—H1A	109.00	C7—C8—C9	128.38 (17)
C10—O3—H3A	109.00	C8—C9—C10	132.05 (17)
C1—N1—C7	128.71 (15)	O3—C10—C9	120.93 (15)
C1—N1—H1	116.00	O4—C10—C9	118.85 (17)
C7—N1—H1	116.00	O3—C10—O4	120.22 (17)
N1—C1—C6	124.70 (16)	C2—C3—H3	120.00
C2—C1—C6	119.82 (16)	C4—C3—H3	120.00
N1—C1—C2	115.48 (15)	C3—C4—H4	120.00
C1—C2—C3	119.73 (16)	C5—C4—H4	120.00
O1—C2—C1	116.18 (15)	C4—C5—H5	120.00
O1—C2—C3	124.10 (16)	C6—C5—H5	120.00
C2—C3—C4	119.91 (17)	C1—C6—H6	120.00
C3—C4—C5	120.47 (18)	C5—C6—H6	120.00
C4—C5—C6	120.35 (17)	C7—C8—H8	116.00
C1—C6—C5	119.70 (17)	C9—C8—H8	116.00
O2—C7—N1	121.78 (16)	C8—C9—H9	114.00
O2—C7—C8	123.78 (16)	C10—C9—H9	114.00
N1—C7—C8	114.44 (15)		
			
C7—N1—C1—C2	−175.7 (2)	O1—C2—C3—C4	179.3 (2)
C7—N1—C1—C6	4.8 (4)	C1—C2—C3—C4	−1.0 (3)
C1—N1—C7—O2	0.0 (4)	C2—C3—C4—C5	0.1 (4)
C1—N1—C7—C8	−179.6 (2)	C3—C4—C5—C6	1.0 (4)
N1—C1—C2—O1	0.9 (3)	C4—C5—C6—C1	−1.2 (3)
N1—C1—C2—C3	−178.76 (19)	O2—C7—C8—C9	2.6 (4)
C6—C1—C2—O1	−179.54 (19)	N1—C7—C8—C9	−177.8 (2)
C6—C1—C2—C3	0.8 (3)	C7—C8—C9—C10	1.2 (4)
N1—C1—C6—C5	179.9 (2)	C8—C9—C10—O3	−4.4 (4)
C2—C1—C6—C5	0.3 (3)	C8—C9—C10—O4	175.8 (2)

Symmetry codes: (i) *x*, *y*−1, *z*; (ii) −*x*+1/2, −*y*, *z*+1/2; (iii) *x*, *y*+1, *z*; (iv) −*x*, *y*+1/2, −*z*+3/2; (v) −*x*+1/2, −*y*+1, *z*+1/2; (vi) −*x*, *y*−1/2, −*z*+3/2; (vii) −*x*, *y*+1/2, −*z*+1/2; (viii) *x*−1/2, −*y*+1/2, −*z*+1; (ix) *x*+1/2, −*y*+1/2, −*z*+1; (x) −*x*+1/2, −*y*, *z*−1/2; (xi) −*x*+1/2, −*y*+1, *z*−1/2; (xii) −*x*, *y*−1/2, −*z*+1/2.

### Hydrogen-bond geometry (Å, °)

**Table:**

*D*—H···*A*	*D*—H	H···*A*	*D*···*A*	*D*—H···*A*
N1—H1···O1	0.86	2.20	2.6111 (19)	109
O1—H1A···O4^i^	0.82	1.94	2.7282 (18)	162
O3—H3A···O2	0.82	1.67	2.4881 (18)	175
C3—H3···O3^i^	0.93	2.47	3.374 (2)	164
C6—H6···O2	0.93	2.26	2.850 (2)	121
C8—H8···O4^vi^	0.93	2.57	3.404 (2)	150

Symmetry codes: (i) *x*, *y*−1, *z*; (vi) −*x*, *y*−1/2, −*z*+3/2.
